# Personal values, subjective well-being and destination-loyalty intention of international students

**DOI:** 10.1186/s40064-016-2439-3

**Published:** 2016-06-14

**Authors:** N. L. Jamaludin, D. L. Sam, G. M. Sandal, A. A. Adam

**Affiliations:** Department of Psychosocial Science, Faculty of Psychology, University of Bergen, Christies Gate 12, 5015 Bergen, Norway; Faculty of Business Management, University Technology MARA, 40450 Shah Alam, Selangor Malaysia

**Keywords:** Destination-loyalty intention, Personal values, Subjective well-being

## Abstract

What are the factors that predict international students’ destination-loyalty intention? This is the main question this paper addresses, using an online survey among 396 (short-term, *N* = 182) and (long-term, *N* = 214) international students at a Norwegian university. Structural equation model-AMOS was conducted to examine relationships among personal values, subjective well-being and destination-loyalty intentions. The results showed that: (1) universalism was positively related to subjective well-being for short-term students; and (2) subjective well-being was positively related to destination-loyalty intention for all groups. We found that relatively stable and happy individuals might be important for ensuring destination-loyalty intentions. Results also indicated that personal values that emphasize justice and equity are also important for short-term international students’ well-being.

## Background

International education is a rising phenomenon worldwide. The internationalization of higher education is one response to the driving force of globalization (Van der Wende 2007). Despite the fact that international students share a number of characteristics with tourists in that both groups are sojourners, very little research attention has been devoted to the possibility of the tourism industry capitalizing on the rising internationalization of higher education for economic gains. Meanwhile, Jamaludin et al. (2016) have argued that international students’ choice of foreign institutions and their loyalty towards these institutions and the host society should be valuable to several stakeholders. Studies have shown that international education generates financial benefits for the host countries (Benos and Zotou 2014; Zhou and Zhang 2014).

Not only do international students serve as ambassadors for their own country during the overseas sojourn, they may also take on another ambassadorial role upon the completion of their studies and stay. They will encourage (or discourage) people in their social network to visit the country (Jamaludin et al. 2016) depending on their experiences during their sojourn abroad. This latter role is linked to the students’ loyalty to the country in which they sojourned, and forms the focus of this study; namely the determinants of international students’ loyalty to the destination where they studied.

Specifically, we examine how international students’ personal values may affect their subjective well-being at their destination, and how these variables (i.e., personal values and subjective well-being) again impact their destination-loyalty intentions. Destination-loyalty intention refers to an individual’s intentions to revisit and recommend the destination to people in their home country (Oppermann 2000; Yoon and Uysal 2005). Moreover, in this study, the interest is on international students’ destination loyalty after their overseas sojourn.

### International students as sojourners

Sojourners by definition are people who travel internationally to achieve a particular goal or objective with the expectation that they will return to their country of origin after the purpose of their travel has been achieved (Safdar and Berno 2016). International students constitute one of the largest and significant sojourner groups (Bochner 2006). As a sojourner group, international students continue to grow in number worldwide, prompting researchers to study their global significance (Safdar and Berno 2016). International students’ contributions to global society and economy are presently well documented, but very little is known about their contribution to the tourism sector. Contemporary international education embraces two categories of students: short-term students who tend to stay for a few weeks to a whole year, mainly to obtain some credits that may be transferred to their home university; and long-term students who often stay for periods longer than a year to complete their academic degree at the overseas university. While short-term students’ sojourns may be longer than those of an average tourist, the longer stay offers them an opportunity to get to know the society better and to develop stronger loyalty intention than the typical tourist. Long-term students may even have a better opportunity to develop destination-loyalty intentions because their overseas sojourn is so much longer accepting that some aspects of international education may involve tourism, it may be economically prudent to understand international students’ destination loyalty and how this may impact global tourism.

### International students as sojourners and tourism

Although there may appear to be superficial similarities between tourists and other sojourning groups, tourism’s unique characteristics contribute to distinctive intercultural experiences and interactions (Safdar and Berno 2016). While literature suggests that relatively few requirements are placed on tourists to adapt to the local host community (Berno and Ward 2005; Mathieson and Wall 1982; Safdar and Berno 2016), Mathieson and Wall (1982) tourists do need to adjust and many of the responses of tourists are not markedly different from those of other sojourners (Hottola 2004; Pearce et al. 1998). Arguing that international students share a number of characteristics with tourists, Jamaludin et al. (2016) have shown that educational experiences of international students impact their loyalty intention to the destination.

Only few studies have to date examined the presumed links between tourism and subjective well-being (McCabe and Johnson 2013). Research suggests that there are significant relationships among personal values, subjective well-being and behavioural intention [i.e., Sagiv and Schwartz (2000), Hallowell (1996), Fornell et al. (1996), Ryu et al. (2010) and Emmons (1991)]. Although links between values, subjective well-being and international students are emerging, consensus on how values may influence subjective well-being and how this in turn may affect destination-loyalty intention remains unclear. The paper highlights this gap and focuses on personal values and subjective well-being.

### Mapping the differences for international students

To gain a better understanding of personal values, Schwartz (2009) suggested categorizing samples according to cultural similarity and dissimilarity with the host society. According to Schwartz (2009), people from Western Europe who are culturally similar to Norwegians score high on egalitarianism, intellectual autonomy, and harmony, and score low on hierarchy and embeddedness. In contrast, people from countries that are culturally dissimilar to Norway such as societies in Eastern Europe, Asia, Africa and the Middle East have cultures that score especially high in affective autonomy and mastery. Schwartz (2009) also suggested that people in the latter cultures tend to find meaning in life through social relationships and obeying expectations from those in roles of greater status or authority.

We argue that these comparisons (i.e., comparing students from culturally similar and culturally dissimilar countries relative to Norway) will increase our understanding of personal values–subjective well-being–destination-loyalty intention among international students. However, a study making such comparisons will require a larger sample size to better understand the significant different between cultures. As the main area of interest for this study is to understand destination-loyalty intention, the present study opted not to dwell very much on cultural differences, as we do not have good measures of culture. Rather, the present study focused more on the duration of the students’ studies (i.e., long-term vs. short term). In a country like Norway, many short-term students come from countries that are culturally similar to Norway, and long-term students tend to originate from culturally dissimilar countries.

#### Duration of studies

There are several reasons for international students choosing an overseas sojourn, and these differ for students who are on short-term study programmes lasting for a few weeks to one academic year, and long-term students who pursue a full degree lasting 2 years or more. A study by Massey and Burrow (2012) found the desire for a cross-cultural learning environment, followed by distinctive academic opportunity, and a unique social experience to be the main motivation of the incoming exchange students. These findings are consistent with previous research such as from Brewer (1983), Carlson (1990) and Sánchez et al. (2006), which found that cross-cultural reasons surpass academic and/or social reasons for studying abroad.

It is known that individuals pursuing exchange programmes for a semester or a year are different from those pursuing a degree that stretches over a couple of years. A number of studies focusing on the motives for studying abroad among long-term students have identified the desire for a cross-cultural experience (Brewer 1983; Carlson and Widaman 1988; Sánchez et al. 2006); and academic and/or foreign language development (Caudery et al. 2008) to be among the principal motivating factors for participating in a study-abroad programme. For short-term students, Massey and Burrow (2012) suggested that the main motivation of the incoming exchange students is a new cross-cultural learning environment, followed by a specific academic opportunity, along with a unique social experience. Acknowledging that short-term and long-term students have different motives for their overseas sojourn, we explored how short-term and long-term students’ personal values may affect their subjective well-being and destination-loyalty intention.

## Reciprocal and causal influences between personal values, subjective well-being and destination-loyalty intention

### Personal values and subjective well-being

Studies by Fischer and Boer (2016), Sortheix and Lönnqvist (2014), Bobowik et al. (2011) and Sagiv and Schwartz (2000) show that the interest in the relationship between personal values and well-being is on the rise. The notion that subjective well-being could and should be used to inform public policy is gaining momentum, and subjective well-being measures have recently been included in government programmes to complement more traditional measures (Diener and Seligman 2004).

Values are motivational goals that influence attitudes, behaviours and evaluations (Fischer and Boer 2016). Schwartz’s value theory (Schwartz and Bardi 2001; Schwartz and Bilsky 1990; Schwartz et al. 2001), describes values as desirable, trans-situational goals of varying importance, which serve as guidelines for action. They influence human behaviour, motivation and goals (Ferssizidis et al. 2010; Homer and Kahle 1988). In other words, personal values reflect what is primarily important to a person and consequently form a central part of an individual’s identity that guides their action. Moreover, well-being can be defined as an optimal psychological functioning and experience that favours both a positive hedonic state and the development of skills and personal growth (Ryan and Deci 2001).

In this study, we took into account both affective and cognitive components of subjective or hedonic well-being, which we considered relevant in relation to personal values and destination-loyalty intention. Hedonic well-being refers both to the prevalence of positive emotions over negative ones and to the level of satisfaction with life and its specific domains (Bobowik et al. 2011). It indicates how people feel and think about their lives (Diener and Scollon 2003).

According to Sagiv and Schwartz (2000), Sortheix and Lönnqvist (2014, 2015), and (Bobowik et al. 2011), a person’s subjective well-being may depend on the person’s value priorities. Thus, we assumed that values could account for some of the predictive variance associated with the destination intentions of international students. Bobowik et al. (2011) nevertheless point out that values are not uniformly related to well-being, with variations across societies being driven by contextual demands in a functional value-fit pattern emphasizing successful adaptation to social and economic demands (Sortheix and Lönnqvist 2014, 2015).

As stated by Sagiv and Schwartz (2000), values that represent growth needs (e.g. self-actualization) become more important when a person attains the goals toward which the values are directed. Growth-need values are those that deal with realizing personal potential, self-fulfilment, and peak experiences (Maslow et al. 1970). Sagiv and Schwartz (2000) further suggested that priority given to growth-related values is likely to correlate positively with subjective well-being. In the personal value model, self-direction, universalism, benevolence, achievement, and stimulation are identified as growth needs (Bilsky and Schwartz 1994). McCabe and Johnson (2013) have suggested that growth needs might include high-involvement tourism experiences, or those linked to personal and spiritual development. Sirgy (2009) has also suggested that tourism goals related to growth needs are likely to contribute more to life satisfaction and positive affect (moods). We believe international students’ personal values are associated with growth needs, and they have the potential to lead to satisfaction in wider life domains (other than leisure needs).

Following Sortheix and Lönnqvist (2014) assertion, we assume that for short-term students, the majority of whom come from culturally similar countries, universalism, benevolence and achievement will be positively related to well-being. We also postulated that self-direction and stimulation would be negatively related to well-being. By contrast, for long-term students, the majority of whom come from culturally dissimilar countries, we anticipate that achievement, self-direction and stimulation promote well-being, but that benevolence and universalism are detrimental to well-being. Results of studies between personal values and well-being are nevertheless inconclusive (Bobowik et al. 2011). For this reason, in the present study, both long-term and short-term groups of students were analysed to further examine whether personal values are associated with subjective well-being in different types of samples of international students.

Based on the empirical evidence and findings, the present study adopts Schwartz’s value theory (Schwartz and Bardi 2001; Schwartz and Bilsky 1990; Schwartz et al. 2001) with focus on self-direction, stimulation, benevolence, universalism and achievement. Schwartz (2012) argued that people with a self-direction value orientation have more independent thought, curiosity and self-respect, whereas people with a stimulation-value orientation are more directed towards excitement, novelty, and challenge in life. Schwartz (2012) also suggested that people with achievement value orientation are more inclined to seek personal success through demonstrating competence according to a social standard and social recognition, and that people with a benevolence-value orientation are more protective and try to enhance the welfare of those with whom they are in frequent personal contact. Finally, Schwartz (2012) suggested that people with a universalism-value orientation are more understanding, appreciative, tolerant, and protective of the welfare of all people and of nature. We posit that for international students, these values may be a strong predictor of well-being.

### Subjective well-being and destination-loyalty intention

Studies indicate that people who are satisfied with life are also more successful and socially active (Diener et al. 2015; Lyubomirsky et al. 2005). According to Graham and Markowitz (2011), life satisfaction influences an individual’s intention to stay at a destination. They suggest that the chances of relocating are high when people are dissatisfied with their location. Moreover, a study by Özdemir (2014) found that high levels of positive affectivity constitute a state of high energy, full concentration and pleasurable engagement with the environment. Negative affectivity refers to a general dimension of subjective distress and unpleasurable engagement, and is identified by aversive mood states such as anger, contempt, disgust, fear, and nervousness (Özdemir 2014). Affective experiences influence a consumer’s behaviour and perception during consumption interactions (Gountas and Gountas 2007). According to Chi and Yang (2015), if a past event was associated with an unpleasant mood, a similar unpleasant mood in a subsequent time period is likely to activate the recall of relevant information (Bower 1981; Forgas 1995), and potentially elicit a behaviour or intention. Bradburn (1969) has suggested that affectivity influences an individual’s outgoing activities. Following the affective response study conducted by Russell et al. (1981) and Pike and Ryan (2004) have suggested that affect usually becomes operational during the evaluative stage of the destination selection process. It can be argued that present moods can influence individuals’ decisions. Thus, affect in the context of tourism appears to be the evaluative element for destination loyalty or at least destination-loyalty intention. Nevertheless, to date, little research has documented the dynamics of subjective well-being (life satisfaction, affectivity)–intention relationship. In the present study both long-term and short-term international students populations are analysed to examine how subjective well-being (life satisfaction and affectivity) are associated with destination-loyalty intention in different types of samples. No hypotheses have been formulated here with respect to these relationships for short versus long-term students because of differences in the underlying motives for their sojourns abroad.

## Methods

### Sample and procedure

This study was approved by the Norwegian Social Science Data Services (NSD). All registered international students at the University of Bergen were eligible to participate in the study. These students were contacted via email to participate by the International Students’ Office in University of Bergen through its database. Data were collected in 2014. A total of 396 students (36.53 %) accepted the invitation and filled out the questionnaire. Table 1 shows the demographic profile of the respondents, which have been separated into long-term (*N* = 214) versus short-term (*N* = 182). In this study, short-term students comprise those who came to study for periods of less than 12 months and were predominantly students from Western Europe (68.7 %), which is culturally similar to Norway. Long-term students who came to study for 12 months and longer comprise students from around the world with a major proportion (more than 35 %) from Africa and the Middle East. In terms of gender: for long-term students, the number of male students (51.9 %) is slightly higher than the number of female students (48.1 %). For all groups, the majority (more than 50 %) were between 20 and 30 years old. Details of the demographic profile of the respondents can be seen in Table 1.

**Table 1:** Demographic profile of respondents

Long-term			Short-term		
	*N*	%		*N*	%
Female	103	48.1	Female	122	67.0
Male	111	51.9	Male	60	33.0
Total	214	100.0	Total	182	100.0
20–30 years	144	67.3	20–30 years	171	94.0
31–40 years	58	27.1	31–40 years	10	5.5
41–50 years	10	4.7	41–50 years	1	0.5
Above 50 years	2	0.9	Above 50 years	0	0.0
Total	214	100.0	Total	182	100.0
West Europe	23	10.7	West Europe	125	68.7
East Europe	18	8.4	East Europe	45	24.7
Latin America	22	10.3	English-speaking	9	4.9
English-speaking	25	11.7	Asia	3	1.6
Asia	38	17.8			
Africa and Middle East	88	41.1			
Total	214	100.0	Total	182	100.0

### Measurement of the variables

#### Destination-loyalty intention

Destination-loyalty intention as our dependent variable was assessed using three items by Oppermann (2000), where two items dealt with revisitation and one item focused on recommending the destination to friends and relatives. The items used were: “After I have completed my course/study, I will travel to Bergen if my financial position permits it”; “My overall feeling about Bergen is so good that I will come again after I completed my course/study”; and “I will recommend Bergen to my friends/relatives as a vacation destination to visit”. Responses were rated on a 5-point scale (1 = strongly disagree to 5 = strongly agree).

#### Subjective well-being

Subjective well-being was assessed throughout the two group samples using an affect balance measure together with life satisfaction. To measure this, the Satisfaction with Life Scale (SWLS) developed by Diener et al. (1985) and the Positive and Negative Affect Schedule (PANAS) developed by Watson et al. (1988) were used.

The Satisfaction with Life Scale includes five items to be answered on a 5-point scale (1 = strongly disagree to 5 = strongly agree). Examples of the questions are as follows: “In most ways my life is close to my ideal”; and “The conditions of my life are excellent”.

PANAS measures positive (PA) and negative affect (NA). The instrument includes 20 words describing different feelings and emotions. The respondents were asked to indicate to what extent they have felt this way in the last 2 weeks. Examples of feelings are: “distressed”; “scared”; “excited”; and “upset”. For the PANAS scale, we assessed affect balance following suggestions drawn from (Watson et al. 1988) and (Diener 2000).

#### Personal values

The 40-item PVQ or PVQ-40 was used in the present study. The PVQ-40 comprises 10 subscales that measure the 10 value types. Each PVQ item comprises a two-sentence short verbal portrayal of a person’s goals or aspirations (Schwartz 2005), e.g. “Thinking up new ideas and being creative is important to him/her”; “He/she likes to do things in his/her own original way”; and “It is important to him/her to be rich.” For each portrait, respondents answered the question “How much like you is this person?” on a 6-point scale (1 = not like me at all to 6 = very much like me).

## Results

Table 2 shows the means and standard deviations for all the scales used in the study. All the mean scores for short-term and long-term were above the neutral point of the scale (i.e. above 3), suggesting that respondents were on the positive side of the scale.

**Table 2:** Descriptive statistics for the variables

	Long term		Short term	
	Mean	Std. deviation	Mean	Std. deviation
Destination-loyalty intention	4.08	0.79	4.17	0.70
Values—benevolence	4.85	0.81	4.64	0.61
Values—self-direction	4.99	0.68	4.95	0.65
Values—stimulation	4.44	1.00	4.28	1.01
Values—achievement	4.26	1.11	4.00	1.11
Values—universalism	5.01	0.69	4.84	0.68
Subjective well-being	4.93	1.51	4.87	1.33

Structural equation modelling (SEM) was used to test the questions arising from the theoretical model. The data analysis was carried out in accordance with the two-step methodology–measurement model and structural model test as recommended by (Anderson and Gerbing 1988).

### The measurement model test

To refine all measures for the structural model, a measurement model using the maximum likelihood estimation method was applied. The initial items relating to three main variables, i.e. subjective well-being (affect, life satisfaction), personal values (self-direction, benevolence, universalism, stimulation and achievement) and destination-loyalty intentions were subjected to a confirmatory factor analysis (CFA). The CFA results on the remaining items showed a good fit to the data. The details of the results are shown in Table 3. The Chi square was also reported to be significant. However, the hypothesized model could be accepted as providing a good fit even though the Chi square value is statistically significant (Anderson and Gerbing 1988), especially with a large sample (Bagozzi and Yi 1988; Hair et al. 2010).

**Table 3:** CFA of measurement model

	Measurement model	Desired model
Long-term		
Chi squared	425.74 (*p* < 0.001)	*p* > 0.05
	*df* = 232	–
GFI	0.85	≥0.90
RMSEA	0.06	≤0.07
TLI	0.88	≥0.90
CFI	0.90	≥0.90
Short-term		
Chi squared	216.07 (*p* < 0.001)	*p* > 0.05
	*df* = 132	–
GFI	0.89	≥0.90
RMSEA	0.06	≤0.07
TLI	0.90	≥0.90
CFI	0.92	≥0.90

A reliability test was conducted to assess internal consistency of multiple indicators for each construct. Details of the results are shown in Table 4. Results in Table 4 indicate that multiple measures in this study are reliable for assessing each construct (Nunnally 1978). However, for the value of average variance extracted (AVE) and composite reliability, which is lower than recommended, the Cronbach Alpha index was evaluated (Baumgartner and Homburg 1996). We noted however that any discussion of interpretation and implication involving the variables with slightly low AVE is provisional and requires replication to further confirm the associations between variables. A construct validity test was conducted using the factor loadings within the constructs, and as shown in Table 4, all standardized factor loadings emerged as fairly high. This showed that the measurement had convergent validity (Anderson and Gerbing 1988).

**Table 4:** Validity and reliability analysis

	Long-term				Short-term			
	Factor loadings	AVE	CR	Cronbach alpha	Factor loadings	AVE	CR	Cronbach alpha
Destination-loyalty intention	0.77	0.73	0.84	0.75	0.78	0.66	0.79	0.68
	0.93				0.84			
Values—self direction	0.62	0.34	0.67	0.67	0.67	0.52	0.68	0.63
	0.60				0.77			
	0.55							
	0.56							
Values—universalism	0.59	0.42	0.78	0.77	0.80	0.46	0.77	0.76
	0.58				0.66			
	0.51				0.53			
	0.78				0.69			
	0.75							
Values—stimulation	0.71	0.41	0.69	0.68	0.69	0.53	0.77	0.77
	0.62				0.72			
	0.62				0.78			
Values—benevolence	0.80	0.48	0.78	0.74	0.71	0.49	0.66	0.47
	0.59				0.69			
	0.82							
	0.52							
Values—achievement	0.69	0.55	0.83	0.82	0.72	0.61	0.86	0.86
	0.85				0.83			
	0.73				0.77			
	0.68				0.79			
Subjective well-being		0.43	0.59	0.78		0.38	0.55	0.78
Life satisfaction	0.51				0.57			
Affect	0.77				0.66			

Finally, results in Table 5 indicate that discriminant validity is well established. Following Hair et al. (2010), no correlation among the latent variables exceeded 0.9, which suggests good discriminant validity. In fact, Table 5 shows that the correlation coefficients among the latent constructs did not exceed 0.9. Therefore, the model is assumed to be free from multicollinearity problems (Fidell and Tabachnick 2006; Hair et al. 2010). From the tests for reliability and validity, strong evidence was found to suggest that the constructs satisfied the requirement for reliability, convergence and discriminant validity.

**Table 5:** Discriminant validity test

	DLI	SD	U	S	B	A	SWB
Long-term							
DLI	0.854						
SD	0.048	0.583					
U	0.050	0.714	0.650				
S	0.068	0.886	0.508	0.651			
B	0.048	0.728	0.874	0.586	0.695		
A	0.055	0.518	0.383	0.588	0.428	0.741	
SWB	0.215	0.222	0.231	0.318	0.226	0.255	0.653
Short-term							
DLI	0.811						
SD	0.149	0.722					
U	0.110	0.091	0.677				
S	0.068	0.333	0.257	0.731			
B	0.728	0.205	0.546	0.387	0.700		
A	0.066	0.374	−0.149	0.312	0.069	0.779	
SWB	0.241	0.068	0.331	0.204	0.331	0.198	0.617

*DLI* destination-loyalty intention, *SD* value—self-direction, *U* value—universalism, *S* value—stimulation, *B* value—benevolence, *A* value—achievement, *SWB* subjective well-being

### The structural model test

The hypothesized model was tested for goodness-of-fit using AMOS 9. Results suggest that for long-term respondents, goodness of fit Index (GFI) = 0.91; root mean square error of approximation (RMSEA) = 0.05; Tucker Lewis index (TLI) = 0.95 and comparative fit index (CFI) = 0.96; and for short-term respondents, goodness of fit Index (GFI) = 0.93; root mean square error of approximation (RMSEA) = 0.04; Tucker Lewis index (TLI) = 0.95 and comparative fit index (CFI) = 0.96; the model was found to achieve adequate fit to the observed data. Thus the proposed structural model satisfies the conditions of unidimensionality.

**Fig. 1:**
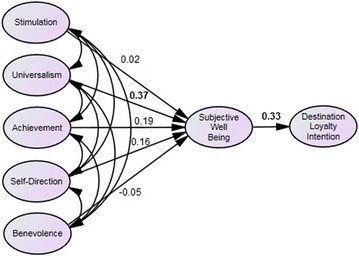
Proposed model on the relationship between values (self-direction, benevolence, universalism, stimulation, achievement) and subjective well-being (life satisfaction, positive affect and negative affect) and their influence on destination-loyalty intention for short-term students. The significant results are depicted in *bold*

**Fig. 2:**
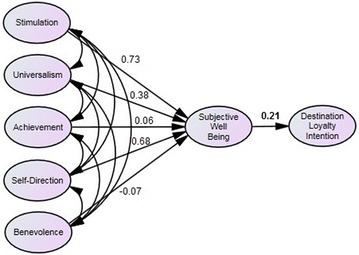
Proposed model on the relationship between values (self-direction, benevolence, universalism, stimulation, achievement) and subjective well-being (life satisfaction, positive affect and negative affect) and their influence on destination-loyalty intention for long-term students. The significant results are depicted in *bold*

For short-term students, results indicate that of the five personal values tested, only universalism is significant (β = 0.37, *p* value <0.05). The other personal values: benevolence (β = −0.05, *p* value = 0.775), self-direction (β = 0.16, *p* value = 0.315), stimulation (β = 0.02, *p* value = 0.904) and achievement (β = 0.19, *p* value = 0.224) were all not significant. With respect to long-term students, none of the personal values was found to be significantly related to subjective well-being, i.e., universalism (β = 0.38, *p* value = 0.344), benevolence (β = −0.07, *p* value = 0.804), self-direction (β = −0.68, *p* value = 0.385), stimulation (β = 0.73, *p* value = 0.307) and achievement (β = 0.06, *p* value = 0.639). However, the relationship between subjective well-being and destination-loyalty intention was significant for both short-term (β = 0.33, *p* value <0.05) and long-term (β = 0.21, *p* value <0.05) students. The results are depicted in Fig. 1 (short-term) and Fig. 2 (long-term).

## Discussion

Our point of departure for this study was that subjective well-being and destination-loyalty intentions may have relevance for public policy in international education. This contention however, turned out not to be as straightforward as we assumed. Many of the relations between personal values, subjective well-being and destination-loyalty intentions turned out not to be significant.

One clear finding from the analysis is the role of universalism in subjective well-being among short-term students. This finding is also supported by the notion that social-focused values (benevolence) promote well-being among West Europeans (Sortheix and Lönnqvist 2014). In other words, short-term international students (mostly exchange students) appear to favour conditions that promote selflessness, understanding, appreciation, tolerance and protection for the welfare of all people and for nature in order to feel good about themselves. Stated another way, international students who benefit from their stay in Norway emphasize the clean nature and the welfare society of the country. Highlighting these aspects of Norway may be important when trying to attract students to come to Norway.

Although we did not find a significant relationship between benevolence and subjective well-being, the result is consistent with Sagiv and Schwartz (2000) suggestion that benevolence values may not necessarily be related with well-being. This value emphasizes caring about the well-being of other people. For exchange students who feel isolated or struggle with their social adaptation, a strong emphasis on social relationships may have negative consequences.

Similarly, our analysis did not show significant relationships between self-direction and subjective well-being. Self-direction refers to an emphasis on independent thoughts and actions (Schwartz 2012). The lack of significant relationship may reflect the fact that autonomy is not an important source of gratification for international students.

The result of stimulation value orientations–subjective well-being also indicates that stimulation is not related to international students’ subjective well-being. The present finding is not consistent with the other studies, i.e. Ryan et al. (1996) and Sortheix and Lönnqvist (2014). It appears that in international education, and for international students in Norway in particular, the conditions of being adventurous, exciting and independent may not be important predictors of their well-being.

Finally, we did not find any significant relationship between achievement-value orientations and international students’ subjective well-being. The finding suggests that individuals high on achievement-value orientation may be less concerned with loyalty to a destination, and their happiness with respect to a destination may be less relevant for both groups of long-term and short-term students.

While this study found very little support regarding the relationship between personal values and subjective well-being, we think it may be premature to dismiss the potential relationship as non-existent. Further studies on these relationships may be needed before firm conclusions can be drawn. Nevertheless, based on our findings, we recommend that stakeholders in the international education industry focus more on universalism as a personal value that enhances subjective well-being for short-term students, to further enhance destination-loyalty intention when implementing regulations, policy and promoting the destination. It is possible that the specific relationship between universalism and satisfaction is specific to exchange students in Norway. Future research should address the extent to which this association iterates among exchange students in other countries.

The second research objectives examined the relationship between subjective well-being and destination-loyalty intention. Here consistent relationships between subjective well-being and destination-loyalty intention were found for both long-term and short-term students, and these are in line with studies by Dagger and Sweeney (2006), Brown and Mazzarol (2009) and Hon and Brunner (2002). Within the context of international education, feelings such as enthusiasm, being active, being alert, having full concentration and pleasurable engagement with one’s surroundings appear to influence one’s intentions to revisit and to recommend a destination.

This finding may have important implications. The significant relationship between subjective well-being and destination-loyalty intention can be further nurtured by education practitioners through adjusting the introduction programs and providing support throughout the sojourn duration. Considering this along with the results of the present study, it seems that interventions targeted on enhancing well-being may influence international students’ destination-loyalty intention, and this may have economic benefits in the long run. We recommend that education practitioners/hospitality managers and marketers consider incorporating into their strategy programs elements that drive international students’ well-being. They should devise strategies for meaningful interactions that embed international students’ in the organization and make them feel like insiders. In particular, education practitioners should make an effort to develop a distinctive service that resonates with their core customers.

## Limitations and conclusions

The study acknowledges the limitations of the approach taken here to analyse the total process of destination-loyalty intention. Perhaps this discrepancy between the results is related to international students following different values that are not tested in this study in relation to well-being and destination-loyalty intention. Generally, our results suggest that some of the relations between personal values and well-being are context-dependent, thereby not supporting models in which the links between well-being and values are qualified by the particular environment (Diener et al. 2003; Sagiv and Schwartz 2000). Future research should consider taking into account the psychosocial situation of the student. The contribution of personal values to well-being (positively or negatively) may depend on whether they are compatible with the values emphasized in the particular environment (Sagiv and Schwartz 2000). We believe the role of personal values needs to be further examined in relation to destination-loyalty intention.

Although the findings of this study help to assure extensive evidence on the relationship between universalism value domains and cognitive/affective aspects of subjective well-being, the insignificant relationship between other personal values with subjective well-being in this study has to be taken into consideration. However, according to Bobowik et al. (2011), values deserve special attention and consideration in research into well-being, because personal growth-related values can indeed make us happier. Thus, Bobowik and colleagues have suggested that in order to examine the relationship in more detail, it may be advisable to include measurements of eudemonic well-being. The eudemonic perspective of well-being may be more strongly associated with value domains (Bobowik et al. 2011). Future research should examine this issue in order to shed more light on the relationship between personal value domains and subjective well-being in international education.

Subjective well-being is a construct consisting of several distinct but related components or dimensions. Future research should also consider the inverse relationship between subjective well-being and destination-loyalty intention. For example, although the subjective well-being to destination-loyalty intention appears to have substantiation in the literature, this relationship is not necessarily straightforward and does not preclude the possibility that there may be a reverse relationship. Broadening this initial line of inquiry in further research on the relationship between subjective well-being and destination-loyalty intention may be translated in the long-term into areas for intervention, allowing efforts to be directed toward creating education destination climates that encourage the development and nurturance of broad expressions of international students’ destination-loyalty intention across various contexts.

We also acknowledge that the data collection, which was limited to only international students in one Norwegian university, may suffer from a single-source bias and generalization of our findings to other countries due to their different national cultures. Further study can also include samples such as culturally similar and dissimilar countries, or perhaps specifically focus on less-developed countries that have not recently experienced rapid social changes to ascertain the validity of the current findings.

Besides, the presented study is correlational in nature. As a potential direction for future research, we proposed that longitudinal studies should be done to help provide clearer evidence of causal relations between personal values–subjective well-being–destination-loyalty intentions.

It is also suggested here that further work on the predictors of destination-loyalty intention is necessary. By extending the proposed model to include other constructs in the relationship between subjective well-being and destination-loyalty intention, i.e., predictors such as personality (Van Oudenhoven and Van der Zee 2002) and stress resilience (Grant and Kinman 2012), further examination can be carried out.

We conclude that universalism values–subjective well-being and personal well-being–destination-loyalty intention in the short term might also in the long run prove to be a good strategy. Our study shows that the happiest countries in the world appear to be those in which universalism values are positively related to happiness and subsequently their destination-loyalty intention. Norway is arguably suited to be considered a model of these.

We argue that the current study provides some insights into the potential for personal values and subjective well-being in influencing destination-loyalty intention in international education experiences. We also believe that our approach could be developed further to offer new insights into research on destination-loyalty intention beyond seeing only personal values (benevolence, self-direction, universalism, stimulation and achievement) and subjective well-being as predictors. Although the relationship between universalism–subjective well-being–destination-loyalty intention represents an important contribution to the understanding of international student loyalty intention, much more research is needed in order to explain relational exchanges in this context, given the present competitive context in which they are now immersed.
